# What do they get out of it? Considering a partnership model in health service research

**DOI:** 10.1017/S1463423621000141

**Published:** 2021-04-08

**Authors:** Anna Askerud, Chrystal Jaye, Fiona Doolan-Noble, Eileen McKinlay

**Affiliations:** 1 University of Otago, Department of General Practice and Rural Health, Dunedin, New Zealand; 2 Department of Primary Health Care and General Practice, University of Otago, Wellington, New Zealand

**Keywords:** collaboration, ethics research, partnership, professional role, qualitative research

## Abstract

A research study to evaluate the implementation of a long-term conditions model of care provoked questions regarding the potential impact of the researcher’s role in health service research. Traditional methods of qualitative interviewing require researchers to be a disembodied presence, objective, and free from bias. When health service research is conducted by health professionals, role conflict may occur if the topic is one they have expertise in, and therefore the ability to provide guidance or information. An alternative perspective to the idea of an independent and objective researcher is the notion of a partnership. In this research collaboration, participants utilised the interview process to reflect and explore different perspectives, and the researcher bracketed their own participation in the phenomenon being studied. Reflexivity was utilised by both participants and the interviewer to ensure transparency and thus bridge the gap between subjectivity and objectivity in qualitative health service research interviewing.

## Introduction

This article discusses the methodology underpinning the conduct of interviews in a current health research study exploring the implementation of a new model of care for those with long-term conditions. As well as being the student researcher, I am also a registered nurse (RN) and a senior nursing lecturer with experience and expertise in long-term conditions management. Tensions between my role as a student researcher and my usual role as RN and lecturer raised some issues of potential methodological conflict in the initial stages of my PhD research project.

## Background

The purpose of the PhD research is to explore health professionals’ experience with a new model of long-term conditions care recently implemented within general practices and Health Care Homes (HCH) across the Southern region of New Zealand (NZ). A mixed-methods methodology is being used with the qualitative component employing a case study approach to understand the experiences of primary care health professionals in general practices of varying sizes and skill mix who were at different stages of implementing the new model of care. Ethics approval was obtained from the University of Otago (Ref: H19/086).

Professional networks and contacts resulted in the first case study, a general practice, agreeing to participate as a pilot site for this research project to test the interview schedule. Agreement was given with the understanding that if this functioned well, the data would be included in the final research analysis. This general practice had only recently implemented the new model of long-term conditions care and the nurses who were leading the implementation had only limited previous experience working within a long-term conditions framework. During the initial research interviews at this field site, the nurses asked me several questions. They were actively seeking advice that would help with the implementation of the new model. I reflected on these interviews and became aware of how an exchange of my knowledge and experience could potentially impact on the implementation of the model of care within this general practice, as well as upon my research. At a subsequent research team meeting, concern was raised as to whether my input into the practice’s implementation journey would influence the data collected, and therefore alter the findings and outcomes of the research study.

It is recognised that making the shift from working as a healthcare professional to performing research presents several challenges (Allen, 2004; Gair, 2012). For example, data collection via semi-structured interviewing traditionally requires the researcher to be neutral, thus reducing bias and interference from the interviewer regarding their own views (Coar & Sim, 2006; Merriam, 2009). This contrasts strongly with the usual nurse role of working collaboratively and in partnership with people for improved health outcomes. The concept of beneficence is also a key nursing value reflecting the concept of contributing to worthwhile outcomes (New Zealand Nurses Organisation, 2019). There is also the issue of ‘insider-ness’ and the degree to which knowledge of context and practice might impact on the researcher’s ability to be a naïve inquirer (Allen, 2004; Gair, 2012). The early stages of this research project raised the question of whether my alter ego as a health professional could be simply ignored in this qualitative health service research.

### Reviewing the paradigms

Epistemology explores how knowledge is gained through research methods and explains how this knowledge can be validated. Modern social science research theories are informed by epistemological stances or paradigms such as constructionism with the defining characteristics of ontology, epistemology, and methodology all arising from the philosophy of knowledge. The ‘way of knowing’ in terms of social constructionism is related to notions of ontology from perspectives rooted in historical, cultural, political, and social viewpoints (Warr, 1999).

From a traditional worldview or paradigm, and to ensure research validity, the qualitative researcher strives to be an objective presence and the interviewer’s role as a participant in the interview process may often not be fully considered in the data generated (Denzin & Lincoln, 2018). Although the interview process may be an opportunity for both researcher and participant to express thoughts and ideas, these aspects are often minimally incorporated into the research report (Sandelowski, 2002). Similarly, while qualitative interviewing is recognised as encompassing influences from the location, time, and the embodiment of the interviewer and how they are perceived by the participant, these constructs have not routinely been incorporated into the resulting research outputs (Denzin & Lincoln, 2018). Thus, the researcher often remains a ‘disembodied’ presence despite acknowledgment of the importance of the socially constructed manner inherent in the qualitative research process (Sharma *et al.*, 2009).

More recently, a growing body of research suggests a reflexive turn in the qualitative landscape, acknowledging the significance of the interviewer as a person and the influence of their presence and their interactions with interview subjects. Initially, feminist researchers began questioning the traditional qualitative stance and objectivity required by the purists in qualitative research (Yow, 1997; Sharma *et al.*, 2009). Subsequently, there has been a shift to more fully acknowledge and incorporate the impact of the researcher’s gender, culture, age, and background, and acknowledge the influence they may have on the process of data collection, the data gathered, and the analysis and reporting of that data. Both Denzin and Lincoln (2018) and Brinkmann and Kvale (2015) are advocates of flexible theoretical paradigms favoured by pragmatists such as Morgan (2007). In a naturalistic constructionist paradigm, the interviewer and participants are interactive and inform each other, and the interview process is a mutually beneficial phenomenon from which both participants may gain knowledge and meaning (Brinkmann & Kvale, 2015).

In health service research particularly, the issue of disclosing your professional self is an important consideration. Gaining insider status may favourably impact on recruitment of participants, as well as affecting the type and quality of data gathered, particularly when interviewing others from your own or other health professional groups. Gair (2012) reviewed the literature on insider/outsider status, which was a term introduced in 1981 by Evered and Louis. The literature suggests that while insider status may be desirable in terms of insight, access, and knowledge, this status may also inhibit critical awareness. Gair (2012) reveals that researchers with either insider or outsider status will both be prone to the complexities of social constructs within the interview process (Gair, 2012).

In an NZ healthcare setting, the Mãori worldview suggests culturally safe practice requires you to share who you are, where you come from, and who your family is. This concept of whānaungātanga, or connectedness, informs a collective approach to health care and is equally relevant in health service research. Relationships in a research setting could be considered a key part of a successful and collaborative research journey, as they are in a successful relationship between a health professional and a patient or client (Barthow *et al.*, 2015; Wepa, 2015).

### A reciprocal epistemology

Historically, there has been debate about the validity of the role of an insider conducting qualitative research (Brinkmann & Kvale, 2015). The use of ‘insider’ status is a pragmatic utility with a shared knowledge and relationships used to the researcher’s advantage. Health professionals conducting research are often working with their peers, utilising existing associations. In addition, insiders who have experienced the phenomenon being studied could potentially compromise the validity of the research. Normative assumptions and practices may go unnoticed, and therefore remain uninspected, by insiders who routinely work within the research setting. Ochieng (2010) deliberately explored her own insider status when interviewing African-American families. She argued that she maintained a dualism in her stance as both an insider and an outsider. Although she gained access to research participants through her own identity as an African American, she believed that she was not considered wholly ‘one of them’ as her identity as an African American was only one aspect of her identity (Ochieng, 2010).

The concept of dualism in my own research was apparent from the beginning. Although I am an ‘insider’ as a health professional, my relationship with these study participants was as both a researcher and a health professional. My existing collegial relationships meant that access to this field site was easily granted, but it became evident during the first interview that these peer relationships and my ‘insider’ status could influence the dynamics of the interview. When the research aims were developed, it was with a clear aim of evaluating a model of care as a researcher. What I had not anticipated was that I might also be viewed by participants as a health professional, and therefore a potential source of information to guide the implementation of the new model of care. One of the nurse participants said to me as I walked in for the first interview: ‘We have more questions for you than you will have for us.’ As a result of these first interactions in the initial case study field site, I began to question the traditional role of the objective researcher utilising interviews to gather data and information from participants. The inequitable researcher role did not align with my primary moral values of caring and beneficence as a nurse (Hoglund *et al.*, 2010). Realising my input could enhance how this practice managed the new model of care, and as a consequence how patients experienced this programme, I felt a duty of care as a nurse and ethically obliged to provide answers to the questions asked. I made a deliberate choice to wear two hats, that of researcher and advisor. Hoglund *et al.* (2010) suggest that to cover both roles and maintain research integrity requires researchers to clearly state the research agenda and establish clear boundaries between the researcher’s potentially dual roles of health professional and researcher during the interview.

Warr (1999) suggests that valid research is about managing both subjectivity and objectivity, and despite the subjectivity inherent in understanding research participants’ worldviews, researchers should use a sound methodology to ensure objectivity, despite this being limited by the subjectivity of participants’ experience. Using bracketing in qualitative interviewing originates in phenomenology and is a method used to reduce bias in qualitative interviewing. Brinkmann and Kvale (2015) describe bracketing as the interviewers attempting to set apart their existing knowledge of a phenomenon to ensure they can be non-judgemental and open to all new information about that phenomenon. Kukkala and Astedt-Kurki (2015) suggest that bracketing in the interview process ensures that the interviewer becomes a repository for the information, rather than being a co-creator despite the social constructs inherent in the process of interviewing. In this study, bracketing was used to separate the different ‘hats’ I wore as the researcher and a health professional colleague. Gathering the participants’ perspective on the implementation journey was ‘on the record’, and during this process, my existing knowledge was bracketed. The subsequent ‘off the record’ part of our interaction was my role as a sounding board and potential resource as a health professional and the brackets were removed.

Harris (2015) in her research into hepatitis discusses the issue of being both a participant and a researcher. Her research draws on ethnographic, phenomenological, and feminist principles and she employs a reciprocal and reflexive attitude to her investigations. Harris shares her own experiences and answers questions pertaining to her journey with hepatitis C. She describes being both a researcher and a participant in the form of a peer educator and explores the uneasy positioning and the ‘messy reality’ of qualitative interviewing (Harris, 2015). The concept of research as a partnership is also discussed by Eide and Kahn (2008) in a health study completed by research nurses. Eide and Kahn (2008) explore the dilemma between maintaining a research ‘stance’ with the ethics of caring (Eide & Kahn, 2008). Barthow *et al.* (2015) discuss the issue of reciprocity with respect to following a Māori research framework. Reciprocity or ‘utu’ is considered an expectation under this NZ model, particularly when information is being provided freely by participants. My interviews with the nurses at the initial field site became a mutually beneficial arrangement. The process of semi-structured interviewing led to the discussion and reflection of issues, which inevitably led to questions from both the participants and myself during the on-record interview. Later, off-record discussions revealed that this process helped the nurses begin to make sense of their situation and clarify the issues they were having with the implementation of the model of care. Although the present research project was developed to evaluate a model of care, all participants gained from the experience through a mutual exchange of information and reciprocity was achieved. There are affinities here with action research where researchers and participants work together to accomplish a common goal for the benefit of the community – in the present case, the community is comprised of primary health professionals implementing a new model of long-term conditions care.

### Is reflexivity the answer?

A key strategy in addressing the influence of professional roles on research outcomes is the art of reflexivity. Reflexivity is the process of ensuring research validity by reviewing and critiquing one’s own experiences and the process of research and considering how this may influence the research outcome (Jootun *et al.*, 2009). Personal reflexivity involves self-awareness and the cognizance of researchers to be both a research device and an individual with social, political, and cultural worldviews. Epistemological reflexivity requires researchers to explore assumptions about the world and about knowledge through the process of conducting research (Dowling, 2006). Researchers are considered a research tool and may have multiple roles over the course of the research, depending on the participants being interviewed. The interview settings can also sometimes allow research participants to explore and work through their own challenges through the discussion process. In this study, reflexivity occurred through my own reflective practice, honed through my nursing experiences. This was combined with research team meetings where I was able to honestly reflect and discuss these experiences and the conflict I felt in the researcher role.

Enosh and Ben-Ari (2016) suggest that participants’ motivation to contribute to research is often fuelled by a desire to be reflective and to consider their own role in the phenomenon under study. Researchers might consider that their research is motivated by the desire to gather data and present academically sound research. This requires investment and curiosity in the topic. Both researcher and participant will therefore bring different agendas to the research process and both agendas must be considered (Enosh & Ben-Ari, 2016). Reflexivity requires a deliberate awareness of actions with researchers being mindful of the construct they create in the environment, and of the self that they present to participants in the research setting. Robust self-scrutiny and awareness of the impact and perspective of both their own role and that of the participant are also important. Reflexivity allows the examination of any agendas present (both personal and professional), which could impact on the data collected. The dialogue in the interview, not just the participants’ responses, must be scrutinised (Enosh & Ben-Ari, 2016; Yow, 1997). Agreeing on a process to separate and delineate the data and any emergent issues that occur during the interview process also requires consideration.

Although ethics are a central tenet in healthcare research, these have often focused on confidentiality, informed consent, and ‘doing no harm’. Honesty through reflexivity is also an important consideration in ethical research (Bishop, 2011). The importance of acknowledging all factors that shape the research, including the impact and disclosure of professional roles, any relationship with the participants, and any input into the phenomenon is important in presenting sound and ethical research. Bishop (2011) also suggests that despite reflexive research practice acknowledging all potential existing social constructs in the process, it is not possible to fully incorporate this in the data gathered because it is not possible to be fully objective within an interview process. Honesty through reflexivity demonstrates the desire and commitment to present sound and ethical research and acknowledges the inherent subjectivity inherent in interviewing.

Glasgow *et al.* (2012) discuss the importance of translational health research for health policy and practice. An evidence integration triangle suggests that an iterative and pragmatic research process should be rapid, practical, transparent, and relevant and that this is essential to ensure that health service research is relevant and easily applied across different settings (Glasgow *et al.*, 2012). Using a collaborative approach in research with health professionals means they can concurrently reflect on their practice, and therefore the research process can be mutually beneficial. While I, as the researcher gained valuable data on the implementation of a long-term conditions programme from a general practice at the start of their implementation journey, participants were provided with a means of exploring and reflecting on their experience of implementing the model of care. Consequently, they had the opportunity to immediately apply any learnings to the ongoing implementation of the model, which could ultimately have positive benefits for patients. In this sense, the research became a reciprocal process with mutual benefits for both researcher and participants.

## Conclusion

Reviewing literature has enabled me to explore different perspectives and experiences in health service research and has led to an understanding and justification for my approach of undertaking off-record and on-record discussions, along with the ability to articulate the methodology required to defend this situation. Combining an objective research stance with a health professional’s duty of care means that the research process and any subsequent discussions where information is imparted must be delineated. An overt research agenda, bracketing, and consistent reflective practice can help to ensure that the interview process with health colleagues is a partnership with mutually beneficial outcomes. In this way, the role of health professional and researcher can be reconciled authentically and both parties in the research partnership can benefit.
